# Heat Shock Protein 70 Is Associated with Replicase Complex of Japanese Encephalitis Virus and Positively Regulates Viral Genome Replication

**DOI:** 10.1371/journal.pone.0075188

**Published:** 2013-09-23

**Authors:** Jing Ye, Zheng Chen, Bo Zhang, Huan Miao, Ali Zohaib, Qiuping Xu, Huanchun Chen, Shengbo Cao

**Affiliations:** 1 State Key Laboratory of Agricultural Microbiology, Huazhong Agricultural University, Wuhan, Hubei, P. R. China; 2 Laboratory of Animal Virology, College of Veterinary Medicine, Huazhong Agricultural University, Wuhan, Hubei, P. R. China; 3 State Key Laboratory of Virology, Wuhan Institute of Viology, Chinese Academy of Sciences, Wuhan, Hubei, P. R. China; University of California, Riverside, United States of America

## Abstract

Japanese encephalitis virus (JEV) is a mosquito-borne flavivirus that causes the most prevalent viral encephalitis in Asia. The NS5 protein of JEV is a key component of the viral replicase complex, which plays a crucial role in viral pathogenesis. In this study, tandem affinity purification (TAP) followed by mass spectrometry analysis was performed to identify novel host proteins that interact with NS5. Heat shock protein 70 (Hsp70), eukaryotic elongation factor 1-alpha (eEF-1α) and ras-related nuclear protein (Ran) were demonstrated to interact with NS5. In addition to NS5, Hsp70 was also found to interact with NS3 which is another important member of the replicase complex. It was observed that the cytoplasmic Hsp70 partially colocalizes with the components of viral replicase complex including NS3, NS5 and viral dsRNA during JEV infection. Knockdown of Hsp70 resulted in a significantly reduced JEV genome replication. Further analysis reveals that Hsp70 enhances the stability of viral proteins in JEV replicase complex. These results suggest an important role for Hsp70 in regulating JEV replication, which provides a potential target for the development of anti-JEV therapies.

## Introduction

Japanese encephalitis virus (JEV) is a neurotropic flavivirus belonging to the family *Flaviviridae*. It is being transmitted to humans through the bite of infected mosquitoes and causes severe central nervous system diseases, including poliomyelitis-like acute flaccid paralysis, aseptic meningitis and encephalitis [1]. JEV mediated encephalitis is one of the most prevalent viral encephalitis worldwide, especially in Eastern and Southeastern Asia. It affects over 50,000 people and results in 15,000 deaths annually [2], with about half the survivors showing neurological sequelae [1].

JEV has a single-stranded positive-sense RNA genome of approximately 11 kb, encoding a single large polyprotein, which is cleaved by the host- and virus-encoded proteases into three structural proteins, capsid (C), premembrane (PrM), and envelope (E), and seven nonstructural (NS) proteins, NS1, NS2A, NS2B, NS3, NS4A, NS4B, and NS5 [3]. Replication of the flavivirus is initiated by a viral RNA replicase complex through a process of RNA-dependent RNA polymerization on the endoplasmic reticulum (ER) membranes. JEV NS3 and NS5 have been identified as major components of the viral replicase complex, which promotes efficient viral replication in close association with cellular host factors [4]. NS5 protein is comprised of two domains, the N-terminal methyltransferase (MTase) and the C-terminal RNA-dependent RNA polymerase (RdRp), which possess enzymatic activities required for capping and synthesis of the viral RNA genome respectively [5]. The presence of flaviviral NS5 is not only restricted to the replicase complex alone, but it’s also free in the cytoplasm and for some flaviviruses in the nucleus of infected cells [6]. The other important functions of NS5 lie in its ability to modulate the cellular pathways and interrupt the host interferon response [7,8], suggesting a crucial role of NS5 in viral pathogenesis.

Viral infection depends on the successful recruitment of host cellular factors at different steps of the viral life cycle, including viral entry, genome replication, viral protein synthesis, viral assembly, and counter defense against cell apoptosis along with innate immunity. Recently, studies have identified numerous cellular factors involved in dengue virus (DV) and West Nile virus (WNV) infection [9]. However, the role of host proteins in JEV infection still needs to be explored completely. In this study, the tandem affinity purification (TAP) approach and mass spectrometry (MS) analysis were performed to screen novel host proteins that interact with JEV NS5. Three host proteins, including heat shock protein 70 (Hsp70), eukaryotic elongation factor 1-alpha (eEF-1α) and ras-related nuclear protein (Ran), were identified. The respective interactions of NS5 with Hsp70, eEF-1α and Ran were further confirmed by co-immunoprecipitation (co-IP) and immunofluorescence assay.

The Hsp70 is a stress-inducible molecular chaperone belonging to heat shock protein family. It consists of a N-terminal ATPase domain and a C-terminal peptide binding domain (PBD) in which there is an EEVD motif enabling it to bind to co-chaperones such as Hsp90, Hsp40 and Hop [10,11]. Hsp70 plays an essential role in the regulation of fundamental cellular processes, including the folding of newly synthesized polypeptides, assembly of multiprotein complexes, transport of proteins across cellular membranes and targeting of proteins for lysosomal degradation. The role of Hsp70 has also been well established in controlling the activity of different regulatory proteins [12-14]. Recently, accumulating studies support Hsp70 to be considered as an important host cellular protein that regulates many aspects of viral life cycle, such as cell entry, genome replication, viral gene expression, viral protein folding, virion assembly, and even virus-induced cell transformation [15-22]. Due to the well-established contribution of Hsp70 in virus life cycle, Hsp70 was selected for further functional assessment. It has previously been reported that Hsp70 is a putative receptor for JEV and is associated with lipid rafts which facilitate JEV entry [23,24]. Here our data demonstrate that Hsp70 directly interacts with RdRp domain of JEV NS5 protein, stabilizes the components of replicase complex and positively regulates JEV genome replication, suggesting a novel role of Hsp70 in the life cycle of JEV.

## Materials and Methods

### Cells and viruses

Human embryonic kidney (HEK293T) cell line and baby hamster kidney (BHK-21) cell line were maintained in Dulbecco’s modified Eagle’s minimal essential medium (DMEM) (Sigma) supplemented with 100 U/ml penicillin, 100 μg/ml streptomycin, and 10% fetal bovine serum (FBS) (GIBCO). JEV P3 strain propagated in BHK-21 was used in this study.

### Plasmid construction

To construct the plasmid encoding the tandem-tagged JEV NS5, the Flag and HA tag were fused in-frame to the 5’ end of the NS5 by polymerase chain reaction (PCR), and then cloned into the pcDNA3.1(+) vector. The plasmids encoding Flag-tagged NS3 or NS5 were generated by cloning the NS3/NS5 gene into the pCMV-Tag1 with the Flag tag sequence fused at the 5’-end of NS5 fragment. The cDNA of human Hsp70 was amplified by PCR and cloned into pCMV-Tag1 with the Myc tag fused at the 3’-end of insert sequence. The truncated mutants of NS5 and Hsp70 were inserted into pCMV-Tag1 generating the plasmids expressing 5’-Flag-tagged NS5 mutants and 3’-Myc-tagged Hsp70, respectively. NS5 RdRp domain (amino acids 406-905), was inserted into pGEX-KG for expression in *E. coli* as a GST fusion protein. All plasmids were confirmed by DNA sequencing. The plasmid carrying the JEV subgenomic replicon fused with a luciferase reporter was kindly provided by Bo Zhang (Wuhan Institute of Virology, Chinese Academy of Sciences).

### Antibodies

Anti-JEV NS3 and NS5 mouse monoclonal antibodies (mAb) were prepared by our laboratory [25]. Commercially available antibodies used include: rabbit anti-Hsp70 polyclonal antibodies (pAbs) (ABclonal), mouse anti-Flag mAb (ABclonal), mouse anti-Myc mAb (Abcam), mouse anti-GAPDH mAb (ABclonal), mouse anti-dsRNA mAb J2 (English & Scientific Consulting Bt.), rabbit anti-K48-polyubiquitin mAb (Epitomics), horseradish peroxidase (HRP)-conjugated anti-mouse and anti-rabbit IgG secondary antibodies (Boster, China), Alexa Fluor® 488 goat anti-mouse IgG (Invitrogen), and Alexa Fluor® 555 goat anti-rabbit IgG (Invitrogen).

### Purification and identification of NS5-interacting Cellular Proteins

HEK293T cells (5×10^7^) were transfected with the Flag-HA-NS5 DNA, or the Flag-HA-vector DNA. At 36 hours (h) post-transfection, cells were harvested with RIPA buffer (150mM NaCl, 1.0% Igepal^®^ CA-630, 0.5% sodium deoxycholate, 0.1% SDS, 50 mM Tris, pH 8.0) (Sigma-Aldrich) added with protease inhibitor cocktail (Roche), and the total cell lysates were subjected to TAP by using the FLAG® HA Tandem Affinity Purification Kit (Sigma-Aldrich) following the manufacturer’s instructions. The purified products were separated by sodium dodecyl sulfate-polyacrylamide gel electrophoresis (SDS-PAGE) and visualized by silver staining. The stained bands were excised, digested in gels with Lys-C, and analyzed by the direct nanoflow liquid chromatography-tandem mass spectrometry (LC-MS/MS) system.

### Co-immunoprecipitation and immunoblot analysis

HEK293T cells (1×10^7^) were transfected with indicated plasmids or JEV subgenomic replicon RNA, or were infected with JEV P3 at 1.0 MOI. At 36 h post-transfection/infection, cell extracts were harvested using RIPA buffer (Sigma-Aldrich) containing protease inhibitor cocktail (Roche). The cell lysate was incubated with indicated antibody at 4°C overnight. 25 μl of protein A+G-agarose beads (Beyotime, China) were added and then incubated for another 3 h. The agarose beads were subsequently washed three times with wash buffer (0.05 M Tris HCl with 0.15 M NaCl). The bound proteins were eluted by boiling with protein loading buffer for 5 min, and then subjected to immunoblot analysis using the indicated antibodies.

### GST-pull down assay

GST-fused NS5 RdRp domain was expressed in *E. coli* BL21 (DE3) cells transformed with pGEX-NS5(406-905). The purified GST-NS5(406-905) or GST protein was mixed with glutathione-Sepharose 4B beads (GE Healthcare) in binding buffer (50 mM Tris-HCl, pH 7.4, 150 mM NaCl, 1 mM EDTA, 1% TritonX-100) for 1 h at 4°C, and the beads were washed with binding buffer. Then the beads were incubated with recombinant His-Hsp70 protein (Sino Biological) for 4 h at 4°C. After washing five times with binding buffer, the bound proteins were separated by SDS-PAGE followed by Western blotting with anti-Hsp70 mAb.

### Immunofluorescence analysis

HEK293T cells were transfected with Hsp70-Myc DNA followed by infection with JEV P3 strain at MOI of 1.0. At 36 h post-infection (p.i.), cells were washed with phosphate-buffered saline followed by fixation with ice-cold methanol. The fixed cells were incubated with the appropriate primary antibodies. After washing, cells were incubated with florescence conjugated secondary antibodies, and then stained 4’, 6’-diamidino-2-phenylindole dihydrochloride (DAPI). The cells were finally washed and observed using a confocal microscope (Zeiss) with 1000× magnification.

### RNA interference

The short hairpin RNA (shRNA) corresponding to the HSPA1A mRNA sequences (5’- TTTCCGGTTTCTACATGCA -3’) (sh-Hsp70) was purchased from GeneCopoeia and used to inhibit endogenous Hsp70 protein expression. Negtive control shRNA (sh-NC) which exhibits no downregulation of any human gene, was also purchased from GeneCopoeia. HEK293T cells at 50% confluence were transfected with 40 nM shRNA plasmids using the X-tremeGENE HP DNA Transfection Reagent (Roche).

### Quantitative real-time RT-PCR

Total RNA was extracted from cells by TRIzol Reagent (Invitrogen) according to the instruction. The reverse transcription was carried out using ReverTra Ace qPCR RT kit (TOYOBO). The level of each cDNA was determined by absolute quantitative real-time PCR using SYBR Green Realtime PCR Master Mix (TOYOBO), and fluorescent signals were analyzed by an ABI StepOne Plus system (Applied Biosystems). The JEV NS5 gene of cDNA samples and standard plasmids were amplified using primer paires of 5’-TACAACATGATGGGAAAGCGAGAGAAAAA-3’ and 5’-GTGTCCCAGCCGGCGGTGTCATCAGC-3’.

### Plaque assay

Cells transfected with sh-Hsp70 or sh-NC were infected with JEV at an MOI of 1.0. At 12, 24, and 36 h p.i., cells were harvested and virus titers were determined by plaque assay in BHK cells. Briefly, viruses were serially diluted and inoculated onto monolayers of cells. After 1 h of absorption, cells were washed with serum free DMEM and cultured in DMEM containing 3% FBS and 1.5% sodium carboxymethyl cellulose (CMC, Sigma-Aldrich). Visible plaques were counted and viral titers were calculated after 3 days of incubation. All data were expressed as the mean of triplicated samples.

### In vitro transcription of JEV replicon and replication analysis

The plasmid carrying the JEV subgenomic replicon fused with a luciferase reporter gene was transcribed in vitro using the T7 MEGAscript kit (Ambion) following the manufacturer’s instructions. The transcribed JEV replicon RNA was transfected into 293T cells using the Lipofectin 2000 (invitrogen). At 12, 24 and 36 h p.i., cells were harvested and the replication of JEV replicon was analyzed by RT-qPCR, Western blot and Luciferase assay (Luciferase Assay System, Promega).

### Immunoprecipitation-RT-PCR

Immunoprecipitation-RT-PCR (IP-RT-PCR) was performed as described by Katoh H, et al. [26]. Cells (1×10^7^) transfected with Hsp70-Myc plasmid were infected with JEV at an MOI of 1.0. At 36h post-infection, cells were harvested with RNA-protein binding buffer (10mM HEPES [pH 7.3], 500 mM KCl, 1 mM EDTA, 2 mM MgCl_2_, 0.1% NP-40, 0.1 μg/μl yeast tRNA, 1 U/ml RNase inhibitor [TOYOBO] and protease inhibitor cocktail [Roche]). The cell lysate was incubated with anti-HA antibody at 4°C overnight. 25 μl of protein A+G-agarose beads (Beyotime, China) were added and then incubated for another 3 h. The agarose beads were subsequently washed three times with RNA-protein binding buffer without yeast tRNA, and RNA was isolated by TRIzol reagent (Invitrogen). RT-PCR was carried out using ReverTra Ace qPCR RT kit (TOYOBO), followed by PCR with La Taq polymerase (Takara) and primer sets (5’-TCTGTCACTAGACTGGAGCA-3’/ 5’-CCAGAAACATCACCAGAAGG-3’) targeted to a fragment consisting of nucleotides 2652 to 3589 in the JEV NS1 gene.

### Ubiquitination analysis

To measure ubiquitination level of NS3 and NS5 *in vivo*, 293T cells were transfected with sh-Hsp70 or sh-NC. At 24 h post-transfection, cells were transfected with Flag-NS3 or Flag-NS5 plasmid or JEV replicon RNA. MG132 (20 nM) was added during the last 6 h of culture. Cell lysates were harvested in RIPA buffer containing 20 mM N-ethylmaleimide (NEM) (Sigma-Aldrich) at 36 h post-transfection. Samples were subjected to immunoprecipitation assay as described above with anti-NS3 or anti-NS5 mAb. Western blot analysis was subsequently performed with anti-NS3, NS5, and K48-polyubiquitin antibodies.

### Statistical Analysis

The student’s t test was used to compare between two treatment groups. *P* values of less than 0.05 were considered as statistically significant. All statistical analyses and calculations were done using GraphPad Prism 5 (GraphPad Software Inc, La Jolla, CA).

## Results

### Identification of host cellular proteins interacting with JEV NS5

**Figure 1:**
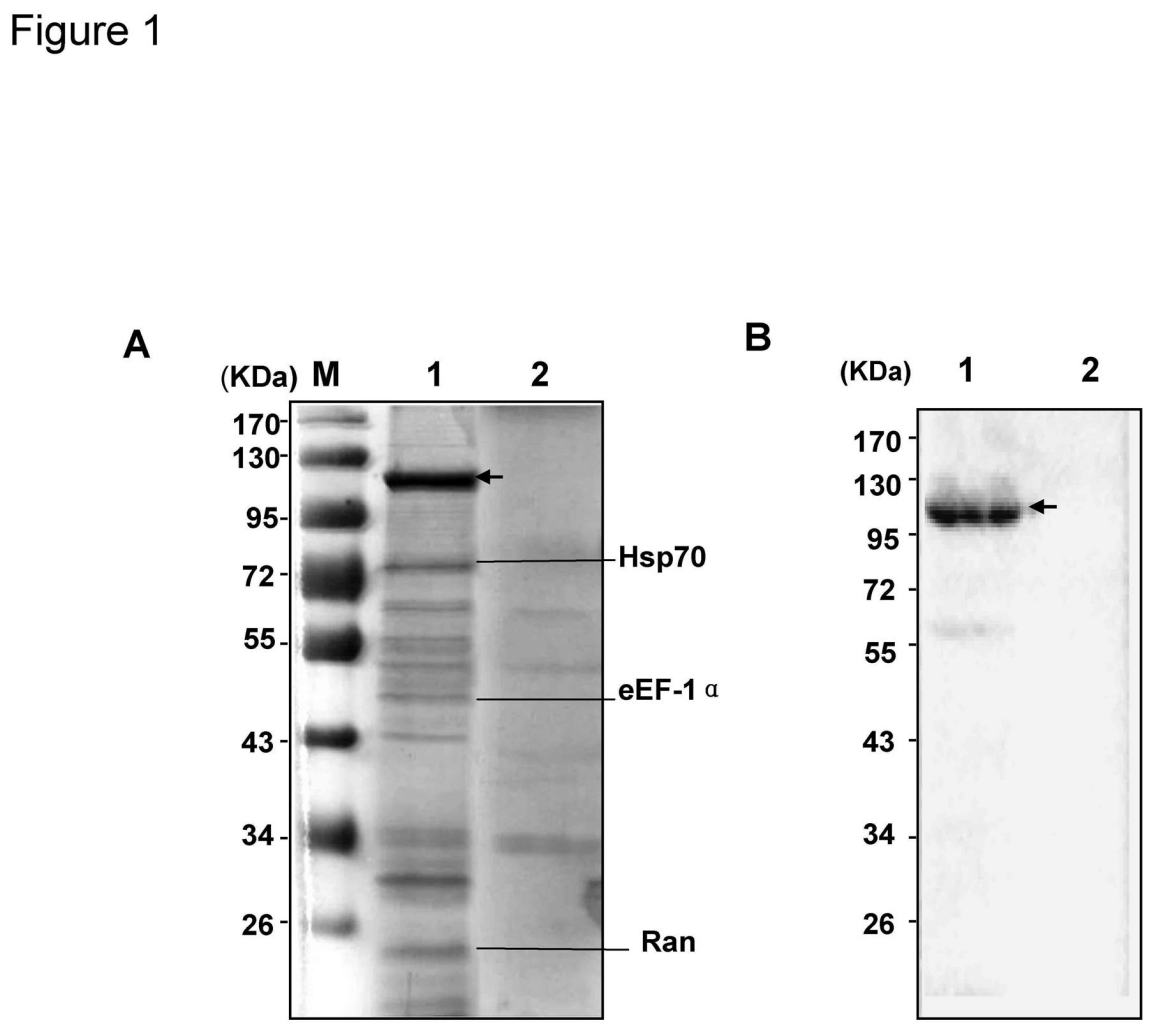
Identification of cellular proteins interacting with JEV NS5. (A) Purification of NS5-interacting proteins using the TAP method. Cell lysates from 293T cells transfected with either the pcD-Flag-HA-NS5 DNA (lane 1), or pcD-Flag-HA vector (lane 2) were purified using the TAP procedure as described under “Experimental Procedures.” The purified proteins were visualized by silver staining. The protein bands in lane 1 which is different with that in lane 2 were excised and analyzed using LC-MS/MS. The position and names of proteins identified by MS are indicated. (B) Western blot analysis of the purified proteins. Anti-Flag antibody was used to detect Flag-HA-NS5 protein. In lane 1 of both panels, the black arrowheads mark the expected full length Flag-HA-NS5.

To identify novel cellular proteins interacting with JEV NS5, TAP followed by LC-MS/MS analysis were performed. The construct containing two tandem tags, Flag and HA, fused to the N-terminus of NS5 was expressed in 293T cells and purified with binding proteins as described in “material and method”. The purified protein complex was separated by SDS-PAGE and visualized using silver staining. A protein band with the molecular mass of about 99KD (consistent with Flag-HA-NS5) along with several co-purified protein bands was observed (Figure 1A). The expression of Flag-HA-NS5 was subsequently confirmed by Western blotting (Figure 1B). The individual co-purified protein bands were excised from SDS-PAGE gel and analyzed by LC-MS/MS system. The amino sequence identification showed three proteins with high hit score matching Hsp70, eEF-1α and Ran, respectively, suggesting a possible interaction of these proteins with JEV NS5.

### Hsp70, eEF-1α and Ran are associated with JEV NS5 protein

**Figure 2:**
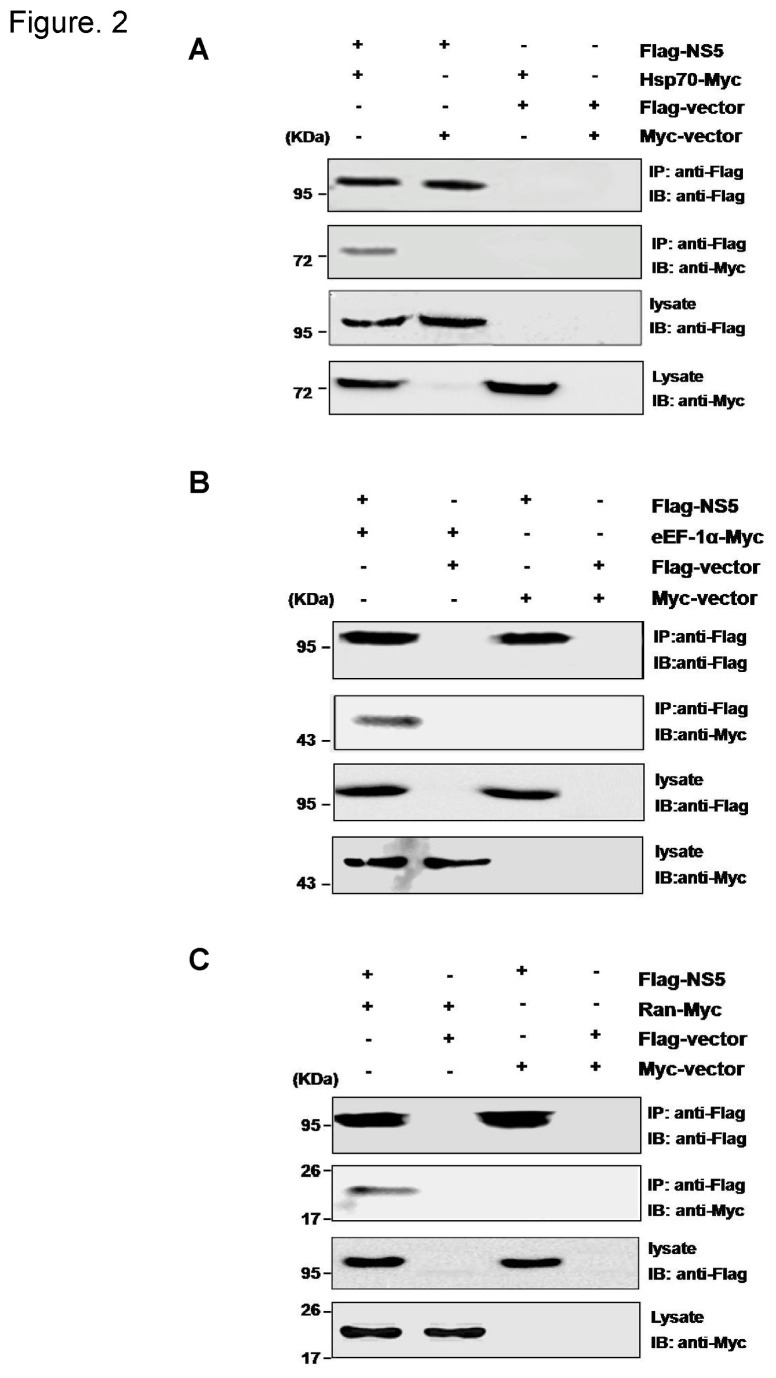
Interaction of the JEV NS5 protein with identified cellular proteins. 293T cells were transfected with expression plasmids encoding Flag-NS5 and Hsp70-Myc (A), eEF-1α-Myc (B) or Ran-Myc (C). Cell extracts were prepared at 36 h post-transfection and used for co-immunprecipitation (co-IP) with Flag-specific antibody. The immunoprecipitates as well as in the cell extracts were subjected to immunoblotting (IB) with anti-Flag and anti-Myc antibodies, respectively. An empty vector was used as a negative control.

The interactions of NS5 with Hsp70, eEF-1α and Ran were further examined by co-immunoprecipitation (co-IP) assay. Lysates of 293T cells co-expressing Flag-NS5 and Hsp70-Myc, eEF-1α-Myc, or Ran-Myc were subjected to co-immunoprecipitation by using specific antibody against the Flag-tag. It was observed that Hsp70, eEF-1α and Ran were co-precipitated with JEV NS5 (Figure 2), confirming the interactions of JEV NS5 with Hsp70, eEF-1α and Ran.

### Hsp70 interacts with JEV NS5 RdRp domain directly

**Figure 3:**
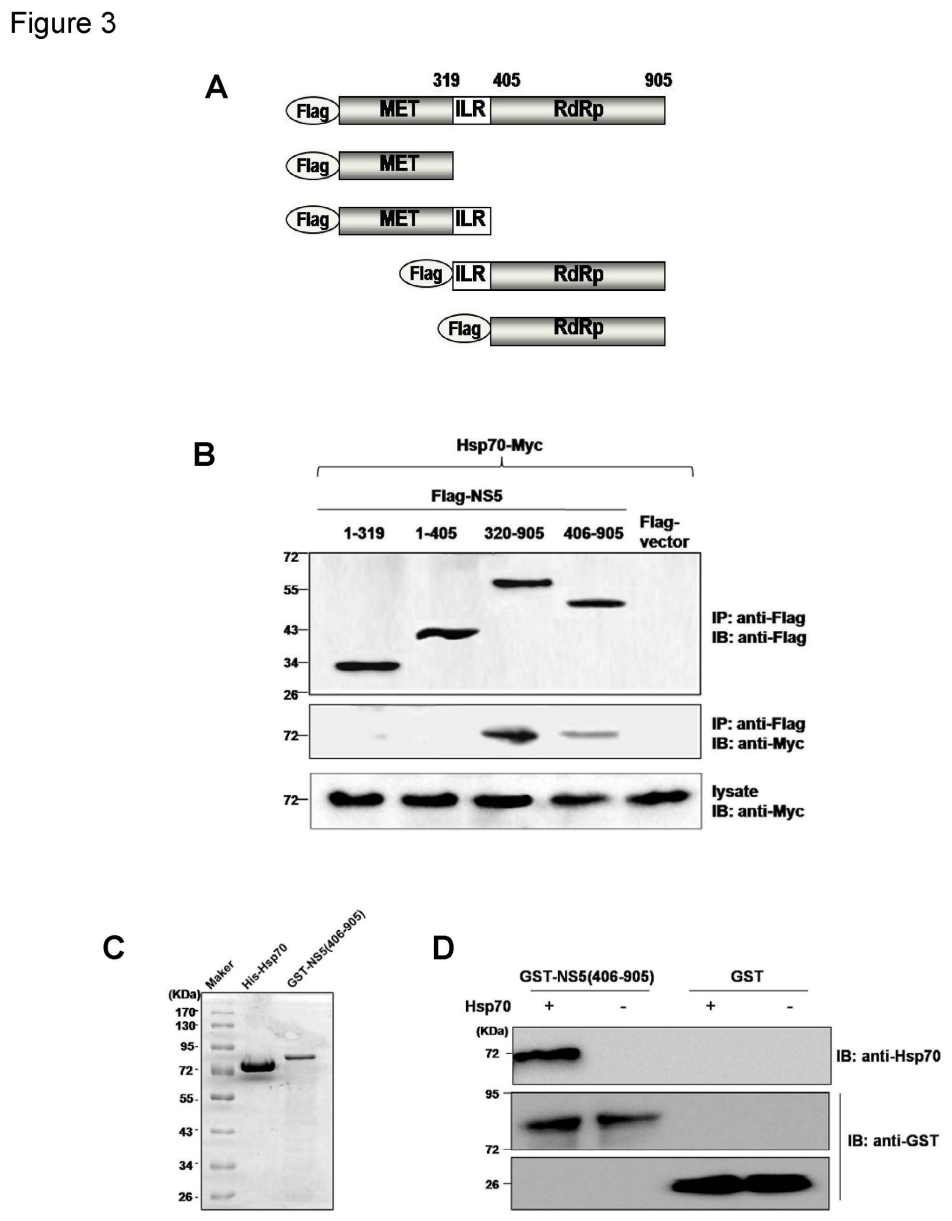
Hsp70 interacts with NS5 RdRp domain directly. (A) The Flag-fused JEV NS5 deletion mutants were generated. (B) Interaction of Hsp70 with deletion mutants of NS5 protein. Hsp70-Myc and a series of deletion mutants of Flag-NS5 were co-transfected in 293T cells, precipitated with mouse anti-Flag MAb and then subjected to Western blotting. (C) The purified proteins of GST-NS5 (406-905) and His-Hsp70 were analyzed by SDS-PAGE. (D) The direct interaction between GST-NS5 RdRp domain and His-Hsp70 was analyzed by GST pull-down assay and Western blotting.

Hsp70 has been known to modulate different infection steps of many viruses including JEV which utilizes Hsp70 as a cellular receptor for viral entry. Since NS5 is one of the key proteins for JEV genome replication, it will be of great interest to observe whether the Hsp70 could also modulate the regulation of JEV replication through interaction with NS5. To understand the significance of this association, we examined which domain of NS5 is required for the interaction with Hsp70. Four NS5 mutants respectively lacking the MTase domain, the RdRp domain, the MTase with internal linker region (ILR) and RdRp with ILR were generated (Figure 3A). Each of NS5 truncated form was co-transfected with Hsp70-Myc plasmid into 293T cells, and the interactions were examined by a co-IP assay. The results clearly showed that the NS5 RdRp domain, from amino acids 406 to 905, but not the MTase domain and ILR, is required for the interaction with Hsp70 (Figure 3B).

To check the direct interaction of Hsp70 with NS5 RdRp, GST pull-down assays were carried out with purified GST-NS5 (405-906) and Hsp70 proteins (Figure 3C). It was shown that the presence of GST-NS5 (405-906) but not GST alone was able to retained Hsp70 (Figure 3D), indicating a direct physical interaction of cellular Hsp70 with the viral NS5.

### Hsp70 interacts with the components of JEV replicase complex

**Figure 4:**
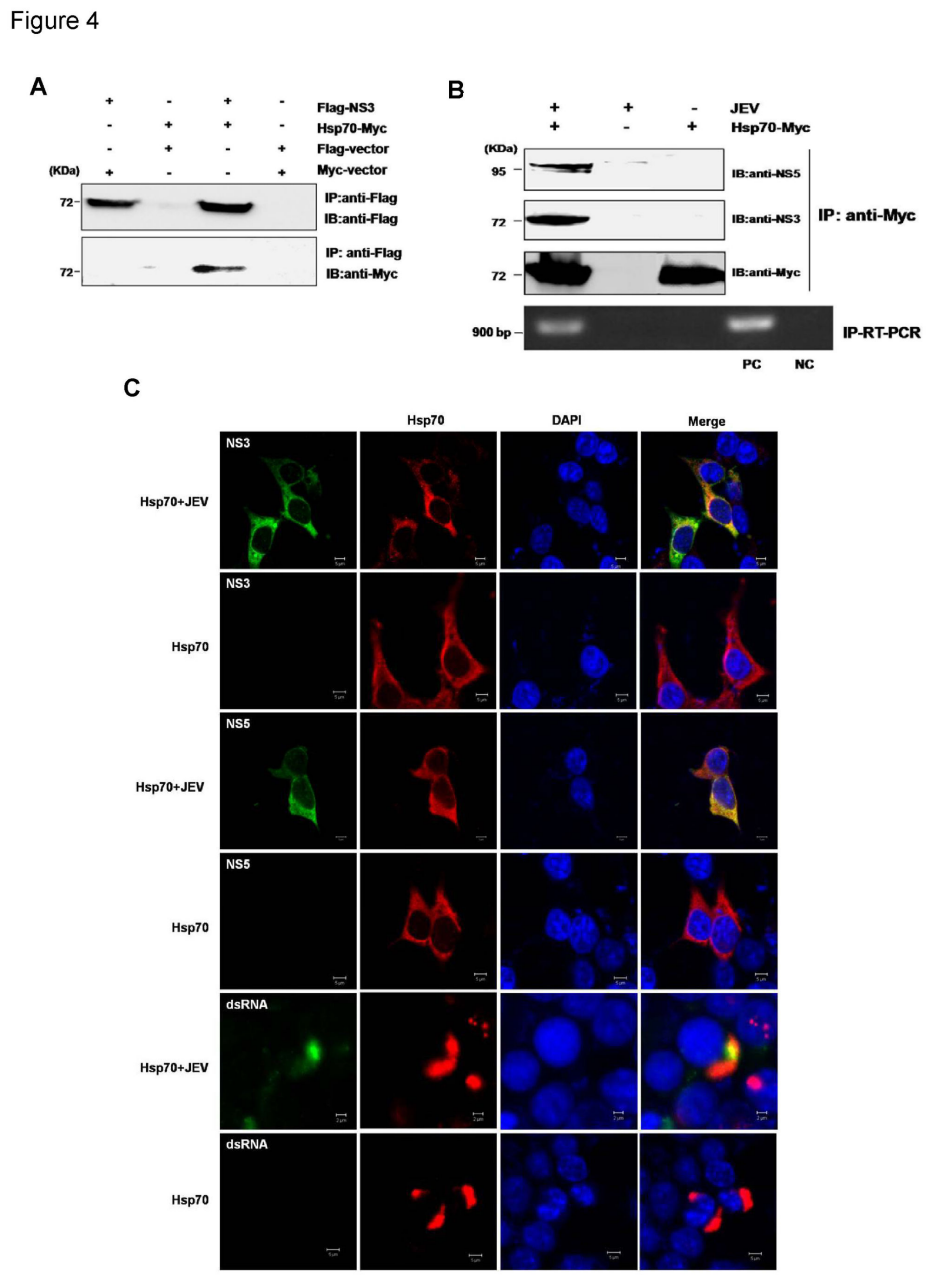
Interaction of Hsp70 with components of the JEV replicase complex. (A) Interaction of Hsp70 with JEV NS3. 293T cells were co-transfected with Myc-tagged Hsp70 plasmid and Flag-tagged NS3 plasmid or vector. Cell lysates were harvested 36 h after transfection for co-IP experiments with anti-Flag antibody. The precipitates were then analyzed by Western blotting with anti-Flag or anti-Myc antibody. (B) Association of Hsp70 with NS3 and NS5 proteins and viral RNA in JEV-infected cells. 293T cells were transfected with Hsp70-Myc expressing plasmid or vector before being mock-infected or infected with JEV at an MOI of 1.0. Cell lysates were harvested at 36 h p.i. and subjected to immunoprecipitation with antibody against Myc, followed by Western blotting for the detection of NS3 and NS5. The RNA were extracted from the precipitates and then subjected to RT-PCR for detecting viral RNA. RNA extracted from cells infected and uninfected with JEV was used as positive control (PC) and negative control (NC) respectively. (C) Colocalization of Hsp70 with NS3, NS5 and dsRNA in JEV-infected cells. 293T cells were transfected with Hsp70-Myc plasmid, followed by mock-infection or JEV-infection. Cells were fixed at 36 h p.i. and were subjected to indirect immunofluorescence to detect Hsp70 (red) and NS3 (green), NS5 (green) and dsRNA (green) respectively. Nuclei are shown by DAPI (blue) staining. The images of cells were acquired with a confocal laser scanning microscope (Carl Zeiss MicroImaging, Inc.).

As described earlier, JEV NS3 and NS5 are major components of JEV replicase complex. A number of studies have revealed a direct association between NS3 and NS5. Therefore, to investigate whether Hsp70 participates in the replicase complex, the interaction between Hsp70 and NS3 was also examined. The 293T cells were co-transfected with Flag-tagged NS3 and Myc-tagged Hsp70 plasmids, and subjected to co-IP assay. Interestingly, Hsp70 was found to be co-precipitated with NS3 by anti-Flag antibody (Figure 4A), suggesting that Hsp70 also interacts with JEV NS3. This result indicates a possible association of Hsp70 with the JEV replicase complex.

It has already been known that the viral replication complex contains not only proteins that are involved in genome replication but also viral double-stranded RNA (dsRNA), an RNA intermediate in replication for several positive-strand RNA viruses including JEV. To validate the association of Hsp70 with the JEV replicase complex, the immunoprecipitation assay and IP-RT-PCR was performed with JEV- or mock-infected cells expressing Myc-Hsp70. The result showed the co-precipitation of Hsp70 with NS3, NS5 and viral RNA in JEV-infected cells (Figure 4B). We further examined the intercellular co-localization of Hsp70 with NS3, NS5 and viral dsRNA in JEV infected 293T cells by immunofluorescence analysis. The strong co-localization of cytoplasmic Hsp70 with NS5 and NS3, as well as dsRNA was observed in JEV-infected cells but not in mock-infected cells (Figure 4C), which confirmed the presence of Hsp70 in the viral replication complex.

### Hsp70 positively regulates the replication of JEV

**Figure 5:**
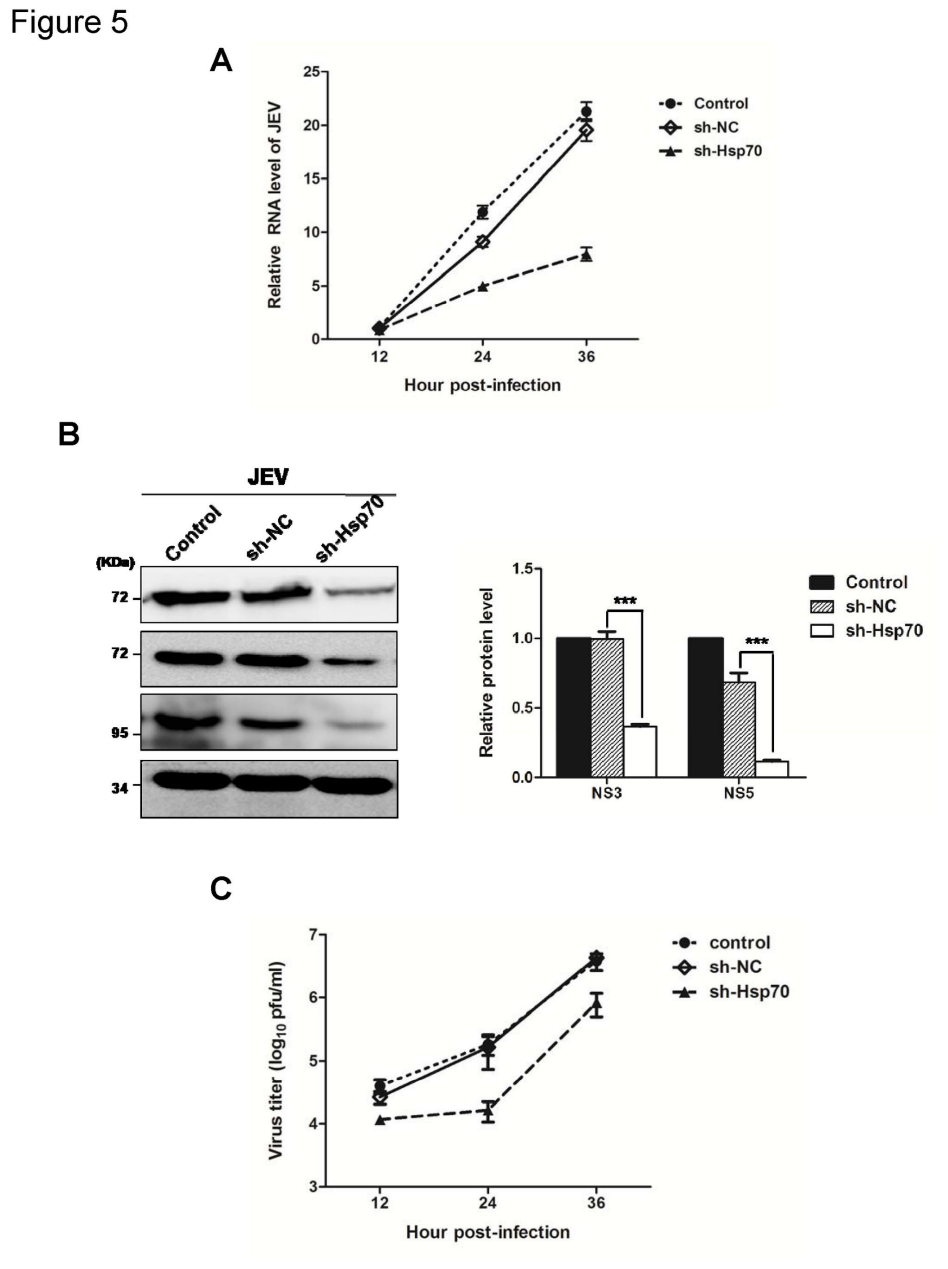
Knockdown of Hsp70 inhibits JEV propagation. 293T cells were not transfected (control) or were transfected with sh-NC or sh-Hsp70. After 24 h, cells were infected with JEV at an MOI of 1.0. (A) Cells were harvested at 12, 24, 36 h p.i. respectively. Total cellular RNA was extracted and subjected to RT reaction. The level of JEV RNA (NS5) was determined by real-time PCR. The results shown are from three independent assays, with the error bars representing the standard deviations. (B) Cell lysates collected at 36 h p.i. were subjected to immunoblotting with mouse MAbs to JEV NS3, NS5, Hsp70 and GAPDH. The protein levels were quantified by immunoblot scanning and normalized with respect to the amount of GAPDH (right panel). The results shown are from three independent assays, with the error bars representing the standard deviations (***, *P* < 0.0001, student’s t-test). (C) Culture supernatants were harvested at 12, 24, and 36 h p.i. and infectious titers were determined by plaque assays in BHK cells. Error bars represent the standard deviations of results from three independent assays.

To understand whether Hsp70 is involved in JEV replication, the cells transfected with sh-Hsp70 or sh-NC were infected with JEV. Then the viral RNA and protein levels were determined by RT-qPCR and Western blotting, respectively. Viral RNA in cells transfected with sh-Hsp70 was reduced by approximately 15%, 46% and 59% at 12h, 24h, and 36h post-infection respectively, compared with that in cells treated with sh-NC (Figure 5A). The viral protein level was also decreased in Hsp70-knockdown cells (Figure 5B). Furthermore, a reduction of viral production in the culture supernatants was observed upon the knockdown of Hsp70 (Figure 5C). These results reveal a positive regulatory role of Hsp70 in JEV infection.

**Figure 6:**
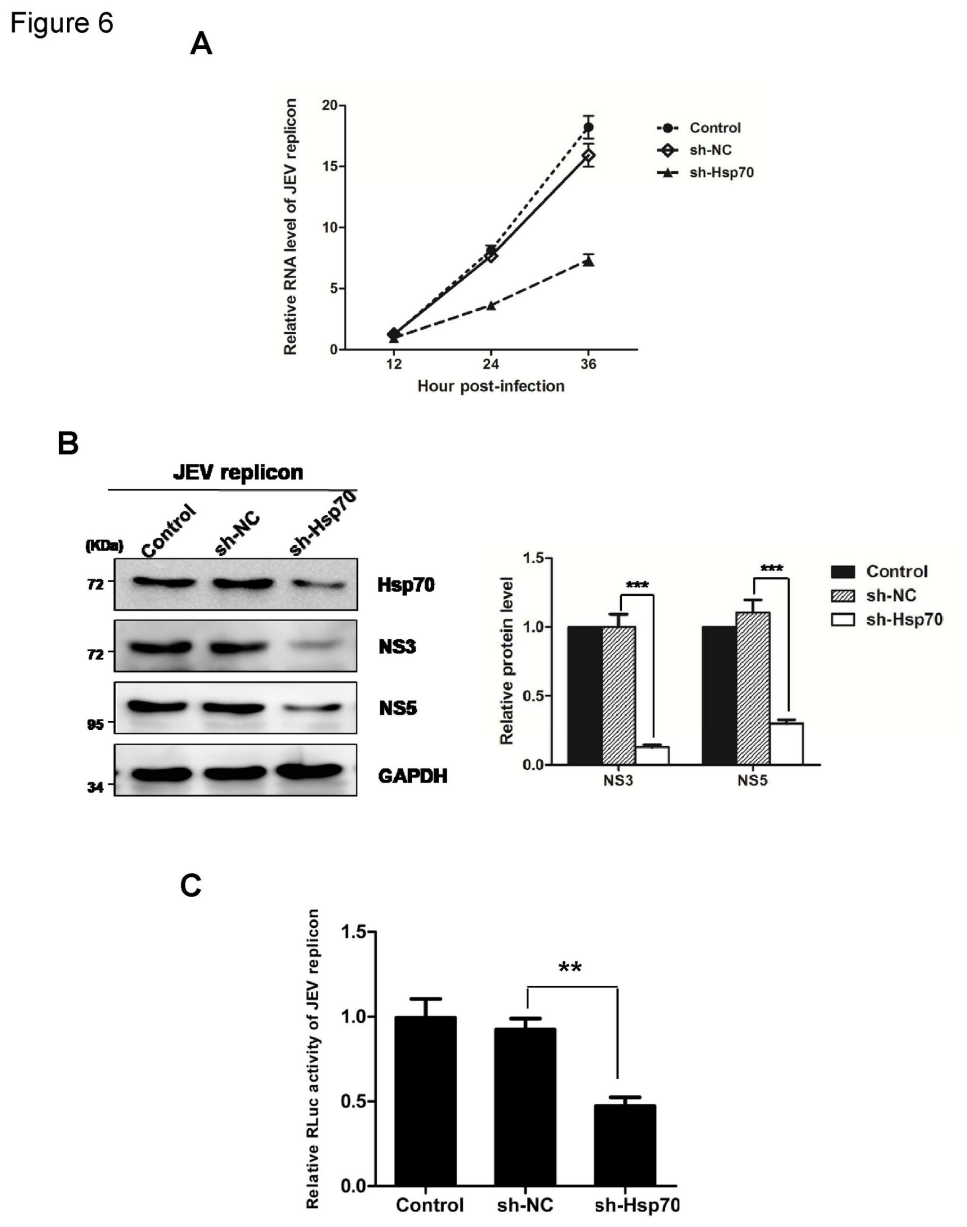
Knockdown of Hsp70 inhibits JEV subgenomic replicon replication. 293T cells was transfected with JEV replicon RNA at 24 h post-transfection with sh-Hsp70 or sh-NC. (A) Cells were harvested at 12, 24, 36 h post-transfection respectively. Total cellular RNA was extracted and subjected to RT reaction. The level of JEV RNA (NS5) was determined by real-time PCR. The results shown are from three independent assays, with the error bars representing the standard deviations. (B) Cell lysates collected at 36 h p.i. were subjected to immunoblotting with antibodies against JEV NS3, NS5, Hsp70 and GAPDH. The protein levels were quantified by immunoblot scanning and normalized with respect to the amount of GAPDH (right panel). Error bars represent the standard deviations of results from three independent assays (***, *P* < 0.0001, student’s t-test). (C) Cell lysates collected at 36 h p.i. were subjected to luciferase assay (**, *P* < 0.001, student’s t-test).

To exclude the effect of Hsp70 on JEV entry and further verify the role of Hsp70 in viral genome replication, cells were transfected with the JEV subgenomic replicon. The results showed that the replicon RNA and proteins were all markedly reduced in Hsp70-knockdown cells compared with that observed in the control cells (Figure 6A and B). Moreover, the activity of the reporter gene Rluc in the replicon was measured by luciferase assay. As shown in Figure 6C, knockdown of Hsp70 inhibited the Rluc activity to about 0.5-fold relative to that of the sh-NC-transfected cells. These data collectively demonstrate that Hsp70 positively regulates JEV genome replication.

### Hsp70 stabilizes the components of viral replicase complex

**Figure 7:**
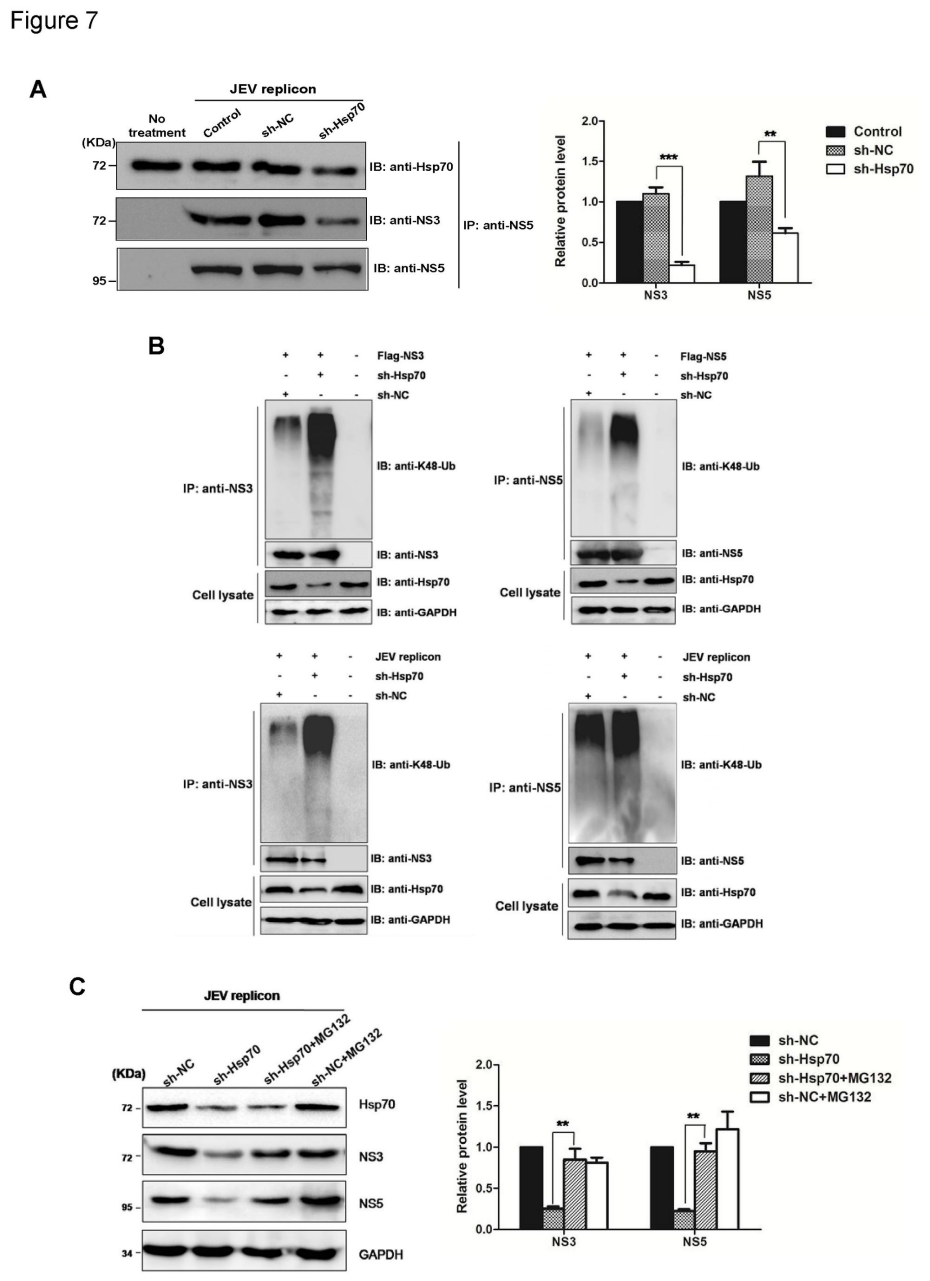
Hsp70 increases the stability of viral proteins in the replicase complex. (A) 293T cells were transfected with sh-NC or sh-Hsp70. After 24 h, cells were transfected with JEV replicon RNA. Cell lysates were harvested 36 h after transfection and immunoprecipitated with an anti-NS5 antibody. The precipitates were subjected to Western blotting with an anti-NS3, anti-NS5, and anti-Hsp70 antibodies. The protein levels were quantified by immunoblot scanning and normalized with respect to the amount of GAPDH (right panel). The results shown are from three independent assays, with the error bars representing the standard deviations (***, *P* < 0.0001, **, *P* < 0.01, student’s t-test). (B) 293T cells containing sh-NC or sh-Hsp70 were transfected with Flag-NS3 or Flag-NS5 plasmid or replicon RNA. Cell lysates were harvested 36 h after transfection and immunoprecipitated with anti-NS3 or anti-NS5 antibody. The precipitates were subjected to Western blotting with anti-NS3, anti-NS5, and anti-K48-ubiquitin antibodies. (C) 293T cells containing sh-NC or sh-Hsp70 were transfected with JEV replicon RNA. In one set of experiment, 20nM MG132 was added at 24 h post-transfection to inhibit proteasomal degradation. Cell lysates were prepared at 12 h after treatment and analyzed by Western blotting with anti-NS3, anti-NS5, and anti-Hsp70 antibodies. The protein levels were quantified by immunoblot scanning and normalized with respect to the amount of GAPDH (right panel) (**, *P* < 0.01, student’s t-test).

As Hsp70 is a cellular chaperone mainly responsible for protein quality control, it is speculated that the recruitment of Hsp70 on JEV replicase complex may stabilize the complex by its chaperone activities. Therefore, the downregulation of Hsp70 may lead to the degradation of replicase complex by proteasome. To validate this hypothesis, anti-NS5 antibody was used to precipitate the replicase complex from Hsp70-knockdown or control cells containing subgenomic replicon. Levels of NS3 and NS5 proteins were checked by Western blotting. The results showed that Hsp70 knockdown diminished NS3 and NS5 proteins in JEV replicase complex (Figure 7A).

We subsequently checked the effect of Hsp70 on the Lys48 (K48)-ubiquitination of NS3 and NS5, which serves as the essential sign of proteins for degradation mediated by the ubiquitin-proteasome system. NS3 and NS5 proteins were immunoprecipitated from Hsp70-knockdown or control cells which express Flag-NS3, Flag-NS5 or JEV replicon, and then subjected to Western blotting with anti-NS3, anti-NS5 and anti-K48-ubiquitin antibodies. It was found that knockdown of Hsp70 greatly increased the K48-ubiquitination of NS3 and NS5 (Figure 7B), suggesting that down regulation of Hsp70 lead to the proteasomal degradation of NS3 and NS5 proteins.

To further validate this mechanism, Hsp70-knockdown cells containing JEV replicon were treated with proteasomal inhibitor, MG132. It was observed that the protein products of JEV NS3 and NS5 were obviously recovered by MG132 treatment (Figure 7C). Taken together, these data suggest that Hsp70 interacts with the viral proteins in replicase complex and protects them being degraded through ubituitin-proteasome system, and thus favor the viral replication.

### JEV infection enhances Hsp70 expression

**Figure 8:**
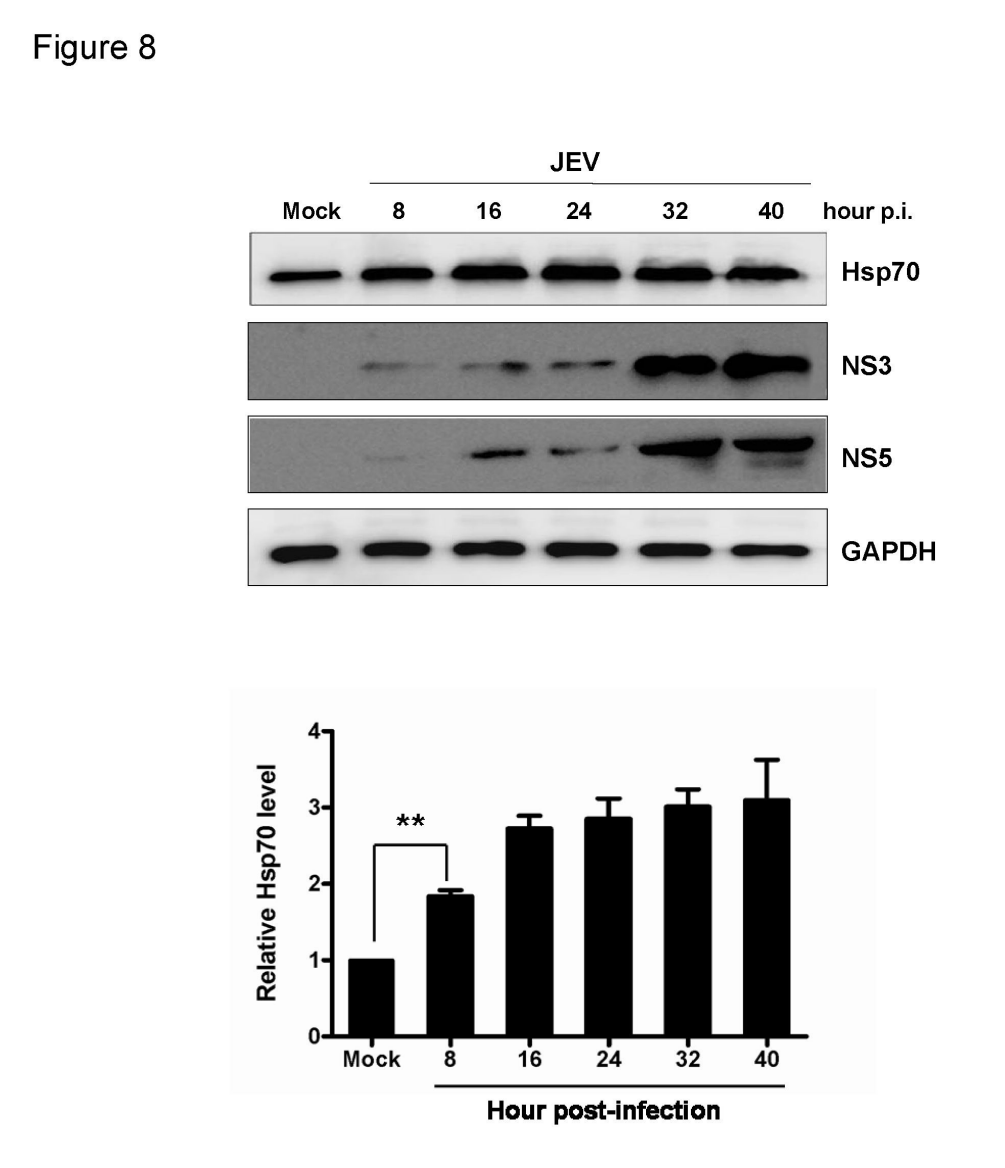
Expression of Hsp70 during JEV infection. 293T cells were mock infected or infected with JEV at an MOI of 1.0. At different times p.i. as indicated, cell extracts were analyzed by Western blotting using anti-Hsp70, anti-NS3, and anti-GAPDH antibodies. The level of Hsp70 was quantified by immunoblot scanning and normalized with respect to the amount of GAPDH (lower panel). Error bars represent the standard deviations of results from three independent assays (**, *P* < 0.01, student’s t-test).

Hsp70 is a stress-inducible protein which can be induced by a variety of stressors like viral infection. To investigate whether JEV infection could affect the expression of Hsp70, JEV-infected cells were analyzed at different time points post-infection by Western blotting. An increased Hsp70 level was observed in JEV-infected cells as compare to control (Figure 8). This data clearly indicates that JEV infection can stimulate the expression of Hsp70.

## Discussion

Within the past few years, much effort on the identification of virus-host cell interactions was spent for complete understanding of viral pathogenesis and the development of novel antiviral therapeutic drugs. JEV NS5 is a key non-structural protein which has an essential role in viral replication and pathogenesis. The process of viral replication in host cells requires the interactions between presumably host and viral proteins along with viral RNA in the replication complex. Recently, JEV NS5 was reported to interact with host cellular proteins, such as Heterogeneous nuclear ribonucleoprotein A2 (hnRNP A2) and Hsp40 which modulate the viral replication [26,27]. In addition to this, JEV NS5 has also been found to inhibit IFN-induced antiviral immune response of host by blocking Jak-Stat pathway [7]. This IFN-antagonistic activity of NS5 is considered to require an intact N terminus of NS5 [8], suggesting a direct interaction of NS5 with host factors probably involved in the mechanism.

To identify new host proteins interacting with JEV NS5 and involved in viral replication and pathogenesis, the TAP approach was employed to purify NS5-interacting complex. This approach has been utilized as an efficient method to purify intracellular protein complexes [28,29]. It enables rapid purification of protein complexes under native conditions and improves specificity greatly in comparison to the results obtained by using the traditional single tag method. By using this method, some human factors were identified. However, not all protein bands studied yielded acceptable identifications and therefore the list of potential NS5-interacting factors is not yet completed.

In this study, human Hsp70, eEF-1α and Ran were firstly demonstrated to interact with JEV NS5. All these proteins have been reported to play important roles in virus infection. eEF-1α interacts with the genomic RNA of various viruses (e.g. WNV, poliovirus and turnip yellow mosaic virus), and modifies the viral replication in infected cells [30-33]. It was also reported to be an essential component of the vesicular stomatitis virus transcriptase complex and was required for replicase activity in vitro [34]. The Ran protein is a small GTPase of the Ras superfamily which is known to have diverse functions in normal cell physiology. One of its major functions is to regulate the nucleocytoplasmic transport of molecules through the nuclear pore complex [35,36]. It has also been found to mediate the nuclear localization of multiple viral proteins including DV NS5 [37]. Since JEV NS5 could interact with viral genomic RNA and was detected in nucleus of infected cells, it is probable that the interactions of NS5 with eEF-1α and Ran might also contribute to JEV replication and nuclear localization of NS5, respectively. However, these remain to be confirmed in our further research. Especially interesting might be the interaction of the JEV NS5 with Hsp70. Accumulating studies have found the critical role of Hsp70 in viral life cycle. Some viruses are inhibited by Hsp70 [15,18-20], whereas some could utilize Hsp70 to help their replication [16,17,38,39]. In case of flavivirus infection, Hsp70 was reported to interact with the E protein on cell surface and function as a putative receptor to mediate infection of JEV and DV. It is also found to be associated with lipid rafts which is required for JEV and DV entry and activation of the phosphoinositide 3-kinase/Akt signalling pathway in the early stage of JEV infection [21,23,24]. Chaperones are also related to cell’s unfolded-protein response (UPR). Induction of the UPR accompanied by up-regulation of GRP78, a member of Hsp70 family, has been widely described for flaviviruses, including JEV, WNV, and DV [40-42]. Recent report suggests that GRP78 may involve in the assembly or release steps of the JEV life cycle [43]. However, as far as could be ascertained, our study is the first one to demonstrate the interaction between Hsp70 and JEV NS5 in cytoplasm, indicating an additional role of Hsp70 in flavivirus life cycle.

The direct interaction of NS5 with Hsp70 was confirmed by GST-pull down assay *in vitro*. In addition to NS5, Hsp70 was also found to interact with JEV NS3. As both NS3 and NS5 are integral components involved in replicase complex of JEV, the association of Hsp70 with the replicase complex and its role in viral replication was further evaluated. It was observed that Hsp70 positively regulates the viral replication. As Hsp70 is a cellular receptor of JEV, this increased viral replication may be attributed by the modified viral attachment and entry. Therefore, the JEV subgenomic replicon was employed to confirm the direct effect of Hsp70 on viral replication. Our results suggest that Hsp70 positively regulates the replication of JEV replicon. These findings are in consistent with the previous reports regarding Hsp70 as a positive regulator for the replication of DV and hepatitis C virus (HCV) [36,38,39], suggesting a probably general role of Hsp70 in family *Flaviviridae*.

During viral infection, a large number of viral proteins are synthesized in a relatively short period of time, which can lead to the protein folding being a limiting step. Therefore, many viruses may need cellular chaperones to facilitate their own protein folding processes. Hsp70, as a central component of the cellular chaperone network, could be utilized frequently by viruses for protein folding. Thus, suppression of Hsp70 expression may decrease the stability of their substrates, leading to proteasomal degradation. In this study, we found that knockdown of Hsp70 increases the K48-ubiquitination of NS3 and NS5 and reduces the their levels in replicase compex, strongly suggesting a proteasome mediated degradation of NS3 and NS5, which may lead to an unstable status of the replicase complex. These results indicate that Hsp70 could stabilize the components of the JEV replicase complex via its chaperone activity, which facilitates the viral replication. This mechanism is similar with that reported in case of HCV infection [39]. Since all the members of genus Flavivirus are closely related, we suggest a possible common mechanism employed by Hsp70 to up regulate the flaviviral replication, but whether it’s a common mechanism in family *Flaviviridae* is still required for further investigation. Hsp40, a co-chaperone associated with Hsp70, has previously been reported to interact with NS5 and aid JEV replication [27], which strongly supports our conclusions. Since they didn’t confirm that the interaction between Hsp40 and NS5 is direct, it is quite possible that this interaction is mediated by Hsp70.

The increase of Hsp70 following viral infection has been widely observed [38,44]. Our results have also demonstrated that JEV infection could induce the expression of Hsp70 from the early stage of infection. This might be due to the rapid accumulation of viral proteins. On one hand, stress-induced increase of Hsp70 accelerates cellular recovery by enhancing the ability of cells to cope with increased concentrations of unfolded/denatured proteins [45]. On the other hand, virus can be benefited from the induction of Hsp70 for its own replication.

In conclusion, three new host proteins (Hsp70, eEF-1α and Ran) that interact with JEV NS5 protein were identified in this study. Further investigation uncovered a positive regulatory role of Hsp70 in JEV genome replication. It was shown that Hsp70 interact with the key components of the JEV replicase complex, and the recruitment of Hsp70 stabilizes the viral proteins in replicase complex. These results can improve our understanding of the mechanism of flavivirus RNA replication and provide a potential target for the development of anti-JEV therapies.
